# Woman on the Wheel

**Published:** 1895-03

**Authors:** 


WOMAN AND THE WHEEL.—In a discussion of the bicycle as a means of exercise, a writer in the *Medical Age* says:

The necessity of maintaining the unstable equilibrium of the bicycle is its chief characteristic. In his work on the 'Physiology of the Bodily Exercises,' Doctor Lagrange states that the best forms, those which assure the symmetical development of the body, are exercises in equilibrium, even the simplest. The bicycle is the most perfect apparatus requiring the maintenance of equilibrium; and itis easy to operate the wheel so that this maintenance may be made more difficult by requiring the more active co-operation of the muscles of the trunk (riding with one hand on the handle-bar, without hands, frequent turnings, etc.).

"The foregoing shows sufficiently my reasons for holding that women will find in the wheel the most perfect means of physical exercise; and experience further confirms this. I do not approve of races for women, however; my observations have simply taught me that wheeling, if kept within wise limits, is highly profitable and will give to a woman a muscular development which many of them sadly need; that it will improve the general health and radically modify many of the forms of *malaise* begotten by a too sedentary life. Let me add, the exercise imparts a certain suppleness, precision and address to the movements, besides cultivating courage and quickness of eye, and may further inspire a salutary curiosity for travel.

"The employment of the wheel introduces new ideas into the life and education. I go so far as to declare that it is a moral agency, since it will permit wife and children to participate in the exercises of the husband and father. Among all the sports it is perhaps the only one which may unite an entire family in the same exercises and pleasures.

